# Convolutional autoencoder based condition monitoring system for unique complex technical systems

**DOI:** 10.1038/s41598-025-26114-w

**Published:** 2025-11-12

**Authors:** Emre Tahtali, Marco Adamscheck, Ludger Overmeyer, Marc Christopher Wurz, Christoph Lotz, Daniel Klaas

**Affiliations:** 1 Institute of Transport and Automation Technology, Leibniz University Hannover, An Der Universitaet 2, 30823 Garbsen, Germany; 2 Institute of Micro Production Technology, Leibniz University Hannover, An Der Universitaet 2, 30823 Garbsen, Germany

**Keywords:** Condition monitoring, Technical systems, Drop tower, Neural network, Data augmentation, Microgravity, Engineering, Aerospace engineering, Mechanical engineering, Information technology, Scientific data

## Abstract

This paper presents the development of a condition monitoring system for a unique, complex technical system represented by a drop tower. The self-developed drop tower, the Einstein-Elevator, is designed as a research platform for highly reproducible zero gravity conditions. As the Einstein-Elevator is a new and unique system with a low number of data samples, detecting wear and other faulty behavior is a challenging task. However, this is important to foresee and counteract cost and time intensive shutdowns. For this purpose, a condition monitoring system based on a neural network approach using acceleration data has been developed together with a framework. According to the state of the art, first the Einstein-Elevator is presented, followed by the developed six-stage framework for model generation. The framework includes both the pre-processing of sensor data as well as the creation and optimization of a data-driven model for diagnosing faulty operating. Additionally, the results showcase the performance of the convolutional autoencoder, which was trained to accurately reconstruct spectrograms of normal flight samples without anomalies. Subsequently, the model was evaluated based on the size of the reconstruction error that occurred with the implementation of anomalous samples. In order to reduce under- or overfitting and improve the model, data augmentation via cutout-methods has been used and validated. This approach resulted in an improved anomaly detection capability as evidenced by several metrics such as the accuracy (97.22%) or the precision (93.88%). Based on the gained research results, the framework has been implemented for the use at the Einstein-Elevator. The work concludes with an outlook for further model optimizations. The developed condition monitoring system approach for a high dynamic system with very precise quality requirements/specifications can be transferred to similarly complex technical systems of various applications, which will be part of future work.

## Introduction

A condition monitoring system (CMS) offers many benefits for operation and maintenance of complex technical systems. Such advantages are for instance an early error detection avoiding expensive repairs, reducing malfunction and downtimes as well as an increased machine life. Mostly, intelligent CMS are used for mass produced machines, which due to their number generate a large amount of data. However, applying such models for complex and unique machines or even whole research facilities is more difficult. Reason for this are often poor data sets and the intention not to create unnecessary wear to obtain the relevant anomalous data. These aspects have a more significant role on CMS based on artificial neural network, as they rely on data. One example of a complex technical system is the Einstein-Elevator (EE) at the Leibniz University Hannover. It is a further development of a classic drop tower. It is designed for experiments under zero gravity conditions with a high quality of the achievable microgravity (< 10^−6^ *g*) and at the same time a high repetition rate of up to 300 experiments per day. It serves as a research platform for various research activities in fields such as physics, production engineering, medicine, biology and materials science^1,2^. Its microgravity quality is key for cutting edge research, which is highly influenced by the high dynamic movement of the EE, and is therefore the driving parameter for drop tower operation and for this research.

Reproducible test conditions are essential for the numerous experiments and test series to statistically evaluate its results. The use of machine learning algorithms from the field of artificial intelligence or the use of findings from the field of advanced methods, such as the use of deep learning models is very promising here^3^. Together with increasingly powerful computers, these are able to process very large amounts of data and extract characteristic patterns^4^. The research question to be answered is whether and how suitable modern machine learning methods can monitor complex systems and facilities such as the EE and whether the existing measurement sensors are sufficient for this. This paper first presents the current state of the art for condition monitoring, particularly with regard to machine learning techniques. The EE and its measurement systems are then described to provide an overview about data recording and its evaluation. This is followed by the methodology regarding the structure of the developed and examined condition monitoring framework. The results are then presented in the following chapter. Finally, there is a discussion about the usability and efficiency of machine learning methods for monitoring unique, complex systems and an outlook on their potential for further development.

### Condition monitoring

In the literature, a distinction is essentially made between three different condition monitoring (CM) strategies. First, there are physical-model-based approaches that describe the CM on a mathematical model^5^. Second, knowledge-based approaches are using expert knowledge. This enables a link between symptoms and associated faults. Third, is the data-driven approach chosen for this work. The performance results purely from a measurement data analysis without knowledge of wear, which is an advantage in comparison to the both former mentioned approaches^6^. Data-based CM consists of the three steps: data acquisition, data processing and the derivation of a maintenance action^7^.

Regarding the first step, the system data is collected with a sensor and stored in a database, as usual. The decisive factor here is to identify usable information from raw data based on knowledge discovery together with the developed database (knowledge discovery in databases (KDD))^8^. In accordance with a vibration-based CMS, which is presented in this paper, an acceleration sensor can be used for recording the vibrations in a system. In connection with this, each system component within a technical system has an individual frequency behavior that is directly related to the operating status. Vibrations due to wear or other faulty behavior generate specific frequency patterns that are included in the sensor data^9^. Using a database containing necessary features is helpful. It enables to obtain a general overview of the system condition, especially when examining the data manually.

The second step is to process the data after it has been recorded. This requires the data to be put into a suitable form in the course of data selection and pre-processing. At this point, exploratory data analysis helps to gain a profound understanding of the data by analyzing it with different evaluation methods, for instance in relation to time and frequency approaches^10^. In reality and connected with the former aspect, data contains errors such as missing data points^11^. It is important to remove inherent measurement errors from recorded measurement data, as a data-driven model is only as good as the associated data basis^12^. Fully prepared data is analyzed and interpreted within the data processing step using appropriate tools. Information that can be used for condition assessment is derived in the form of characteristics; so-called features extracted from the data^7^. For conventional machine learning techniques such as support vector machines or decision trees, these features are extracted in a complex manual process using three different methods based on the time domain, frequency domain or a combination of both^13^. As part of an analysis of the time domain, characteristic features are extracted directly from the temporal course of the data as descriptive statistical features such as the mean value. Alternatively, the analysis can be performed on the signal transformed into the frequency domain. Spectral analysis based on a fast Fourier transform (FFT) is the most proven method. By splitting an oscillation into its individual frequency components, significant frequencies can be identified in the frequency range and analyzed in isolation^7^.

Instead of averaging the frequency content by applying an FFT over the entire duration of the signal, the frequency response can be analyzed using a short time Fourier transform (STFT). Within this so-called time–frequency analysis, the frequencies of a signal are represented as a function of time as a two-dimensional distribution (time–frequency domain)^14^. In such a spectrogram, error patterns are decoded over the course of time^7^. The transformation process of raw data into features and their reduction to a quantity relevant for the maintenance system is summarized as feature engineering^15^.

In the final third step of a data-driven CM concept, maintenance decisions are made on the basis of fault diagnoses^7^. The diagnosis of an abnormal system status is not synonymous with a system failure, as the system components can usually continue to be operated for a short time despite the fault. The function of diagnostics is to detect and identify such errors to signal deteriorating system behavior^15^.

### Neural network-techniques used for feature extraction

The use of neural network methods is very promising for CM, feature extraction and anomaly detection. Here, the model learns to recognize a pattern from data. Algorithms build a statistical model from data sets for this purpose. Deep learning is a subgroup and aims to learn hierarchical relationships in data^16^. The direct difference to purely machine learning is that the features are automatically recognized by the deep learning model and human intervention in the decision-making process is no longer necessary.

One method of deep learning is the use of artificial neural networks. The subfunctions are also referred to as layers; the more layers a neural network has, the deeper it is. Convolutional neural networks are a form of neural network used by autoencoders. This is an encoder-decoder model that is trained to compress input data, minimize it to as few features as possible and then restore the input data from the compressed version. This is particularly suitable for detecting hidden patterns in unlabeled data. The adaptive encoder network transforms high-dimensional data according to Eq. 1 into such an inherent feature representation^17^. Subsequently, an equivalent opposite decoder network maps the features according to Eq. 2 into the original representation as output. The model parameters Θ = [W, b, W′, b′] are optimized by minimizing the reconstruction error between the input and its reconstruction^16^.

1 $$h = \phi { }\left( {{\text{Wx }} + {\text{ b}}} \right)$$

2 $${\text{z }} = \phi \left( {{\text{W}}\prime {\text{h }} + {\text{ b}}\prime { }} \right)$$

Here h stands for the activation of the neuron, z for the reconstructed output vector of an autoencoder and x for the input variable. ϕ describes the existing non-linearity. In addition, W and W’ are the weighting matrices of a neural network and b and b’ are the bias vectors of the encoder/decoder network.

Such techniques are usable for data feature extraction from a variety of technical and complex systems. Within this paper, a drop tower is exemplarily used. To understand its working principles, the drop tower’s basis function and operational setup is described later. For the monitoring of the drop tower a convolutional autoencoder is used.

Convolutional Autoencoders (CAEs) have proven to be crucial in anomaly detection, particularly with limited data sets^18–20^. Techniques such as transfer learning, synthetic data augmentation, and regularization can improve CAE performance^21–24^. By combining these techniques, CAEs can accurately detect anomalies and remain robust and adaptable. CAEs can be applied in various fields such as image analysis and time series analysis^25,26^. Anomaly detection is a crucial component of data analysis, particularly in identifying unusual patterns in time series data. Deep learning methods have proven promising in anomaly detection in multivariate time series. Key approaches include forecasting methods, reconstruction methods, and contrastive methods. Forecasting methods use historical data to predict future values and detect anomalies based on prediction errors. Reconstruction methods use latent representations of the entire time series to detect anomalies. Contrastive methods calculate similarity or dissimilarity between data points to detect anomalies. A comprehensive taxonomy for anomaly detection in multivariate time series was presented by Fengling Wang et al.^27^. Anomaly detection can be applied in various learning paradigms, including unsupervised, semi-supervised, and self-supervised learning. Research in this area is active and constantly evolving^28^.

## The Einstein-Elevator

**Fig. 1:**
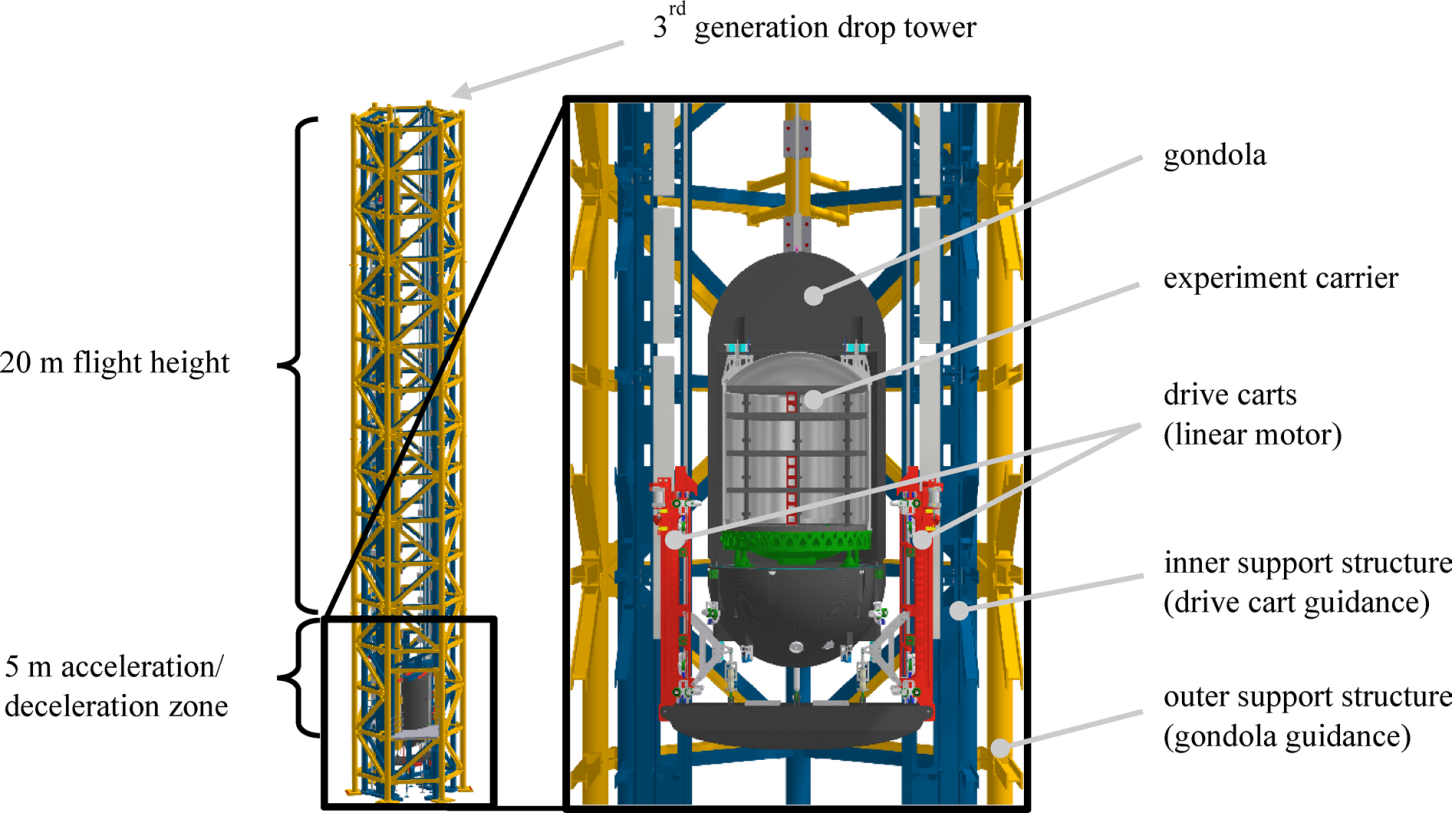
The Einstein-Elevator and its main components.

The EE is a unique, complex system with various data acquisition systems. It is a novel drop tower primarily used for space research located at Leibniz University Hannover. As a consequence of its design, it generates microgravity with a quality of 10^–6^ *g* as well as other gravity levels such as those on the Mars or Lunar surface^29,30^. The facility is able to perform 300 flights per day (100 flights per 8 h-shift operation). There are three main components which play a crucial role to enable these conditions: the drives, the guidance system and the gondola. The gondola contains the experiment carrier within a vacuum atmosphere (Fig. 1).

**Fig. 2:**
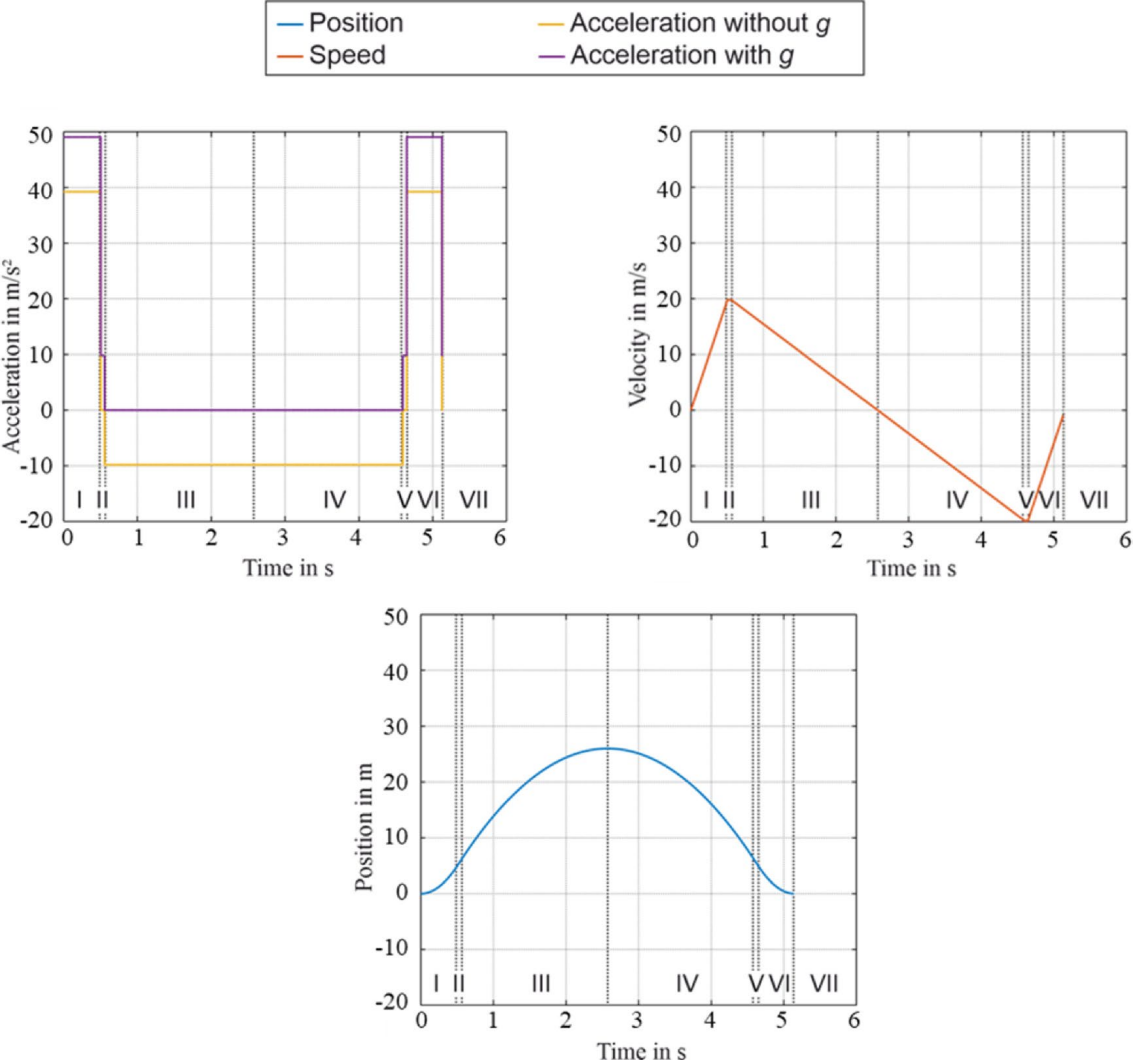
The necessary trajectory profile to enable microgravity conditions. The accelerations (top left), velocity (top right) and the position (bottom); based on ^29^. The applied acceleration to achieve microgravity is shown with and without *g.* Although the absolute acceleration during an experiment (section III-IV) is − 9.81 m/s^2^ (yellow), the relative acceleration is at 0 *g* due to Einstein’s equivalence principle (purple).

If microgravity is simulated, the linear motors accelerate the gondola with 5 *g* (≈ 50 m/s^2^, *g* = 9.81 m/s^2^) for 0.5 s (Fig. 2) with a following short deceleration phase. The deceleration phase creates a distance between the carrier and the gondola (compare section II in Fig. 2). Afterwards, the experiment carrier that contains the experimental set-up is decoupled from the gondola base resulting in a microgravity-period of 4 s (section III + IV). With the end of the microgravity time (section V), the distance of the carrier and the gondola is reduced again back to zero. In section VI, the deceleration of the gondola starts with 5 *g* for 0.5 s and the gondola is arriving at its launching position. At the end of this process, the experiment carrier is centered on the gondola floor because of displacements caused by the Coriolis forces during the free fall time. An important aspect during the motion and in general resulting by the developed structure of the EE is that the gondola and the drive guidance are separated due to the tower-in-tower design. Thus, the transmission of vibrations caused by the motors into the gondola is reduced. The only connection of these two components is the coupling rod. Furthermore, the foundations of the two towers are separated from each other, so that the transmission is reduced even more. A further component which reduces the influence of residual vibrations is the formerly mentioned experiment carrier. It is designed as a pressure vessel and enables a vacuum environment inside the gondola^31^. Consequently, experiments can be conducted under atmospheric pressure inside the carrier while an acoustic decoupling is given. Therefore, the experiment carrier should be used to achieve a high microgravity quality. It also has to be considered while designing the experiments with respect to the load. In this context, the gondola can be loaded with a total weight up to 1,000 kg for experiment setups with 2 m of height and a diameter of 1.7 m. Deviating from the microgravity flights, the experiment carrier has to be attached to the ground of the gondola for desired gravity-levels between μ*g* to 1 *g* (hypogravity) and 1 *g* to 5 *g* (hypergravity). In connection with this, the trajectory of the gondola has to be adapted for the required accelerations, which also influence the duration of the test-time. Another option is to conduct experiments from the top starting position of the tower. As a result, acceleration sensitive experiments can avoid the initial disturbances of the 5 *g*-acceleration phases. This leads to smoother transition from 1 to 0 *g* but reduces the microgravity time to 2 s.

**Fig. 3:**
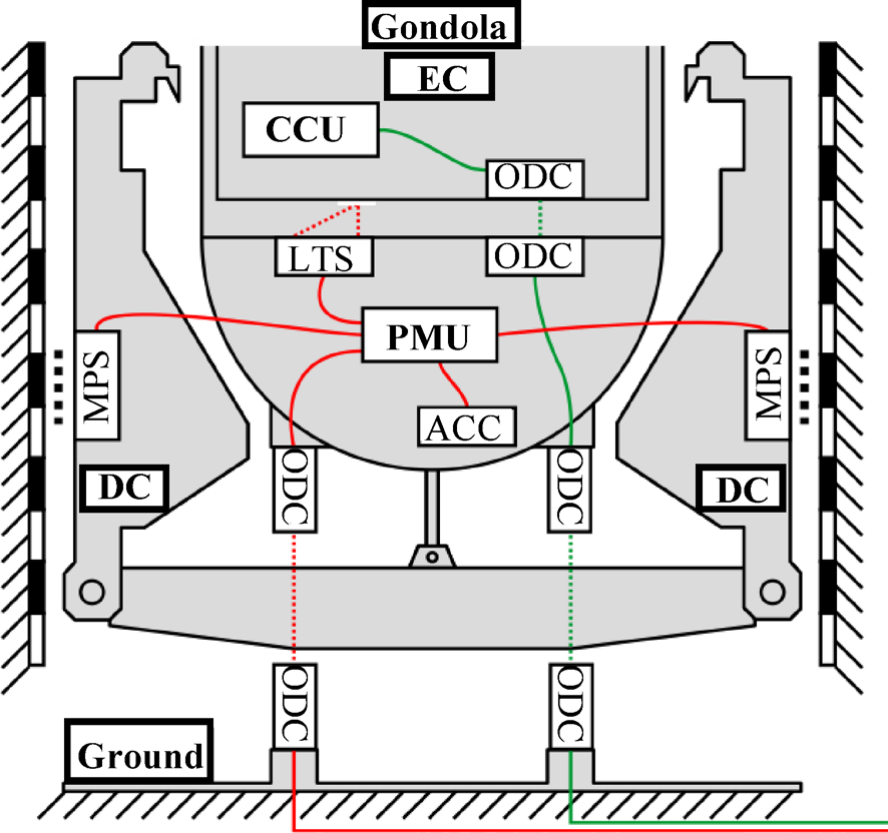
Measurement units and components of the Einstein-Elevator. Acceleration Sensor (ACC), Carrier Control Unit (CCU), Drive Carts (DC), Experiment Carrier (EC), Laser Triangulation Sensor (LTS), Magnetic Position Sensor (MPS), Optical Data Coupler (ODC), Position Measurement Unit (PMU); based on ^29^.

Regarding the data acquisition, the EE has two measurement units: the Carrier Control Unit (CCU) and the Position Measurement Unit (PMU) (Fig. 3). Due to the complexity of the system, those measurement units consist of various sensors which evaluate the recorded data in real-time (sampling rate of 10 kHz), and send their information via an optical data coupler (ODC) to the control room^32^.

The CCU consists of several sensors and a computer system, which records multi-axis data for acceleration, magnetic field, rotation, pressure and humidity through various sensors inside the experiment carrier. Subsequently, it communicates the state of the experiment-carrier. Especially, the acceleration sensors play a crucial role by measuring the quality of the achieved gravity inside the carrier. Therefore, four acceleration sensors are used, which are suitable for different acceleration ranges: two measure accelerations of ± 50 *g* and the others measure accelerations of ± 2 *g*. However, these sensors can’t be used for building a neural network-based CMS due to the reason that they are in free fall and collect data regarding the microgravity quality. As a result, vibrations induced by the outer structure and systems (e.g., the drive system) are isolated (by purpose for the experiments) and therefore not recordable by this measurement system.

In contrast to the CCU, the PMU detects the conditions outside the gondola. It is connected to several sensors:

- An acceleration sensor, which tracks the accelerations of the gondola as well as the vibrations caused by the motion. It is attached outside of the gondola.
- Magnetic position sensors (MPS), which measure the positions of the drive carts.
- A pressure sensor which records the vacuum condition.
- Laser triangulation sensors, which monitor the distance between the experiment carrier and the gondola floor (LTS).

Of all sensors, the acceleration sensor of the PMU is the one, that will be initially used for development of the CMS of the EE. The reason for this is that vibrations of the structure, guidance and motors are directly recorded by the sensor. This is due to its location outside of the gondola.

## Condition monitoring framework

As the EE is a new research facility, it is assumed that there is initially no wear or other damage considering the system. However, due to the reason that it is used frequently, the probability of wear occurring will get higher over time. Therefore, an intelligent CMS is crucial to reduce shut down times as a result of unpredicted damages as well as to maintain the quality and standards of the facility. In this context, another long-term aim is to quantify wear parameters and their influence on key operational parameters such as the microgravity quality.

Considering the fact that there is no labeled data and the formerly assumption of a healthy state of the EE, it is possible to train a deep machine learning model based on a one-class classification (OCC) approach. This enables the detection of faulty system behaviors as anomalies deviating from the normal state^33^. For this purpose, a CMS using a convolutional autoencoder is proposed which is developed to train the normal system state. 

Unlike traditional data-driven machine learning models, deep learning models have the ability to automatically extract latent patterns within data as state features. While models such as recurrent neural networks (RNNs) and long short-term memory networks (LSTMs) are ideal for sequential data and variational autoencoders (VAEs) are used for probabilistic modeling, convolutional autoencoders are particularly advantageous due to their excellent suitability for recognizing and processing spatial and structural features in spectrograms.

**Fig. 4:**
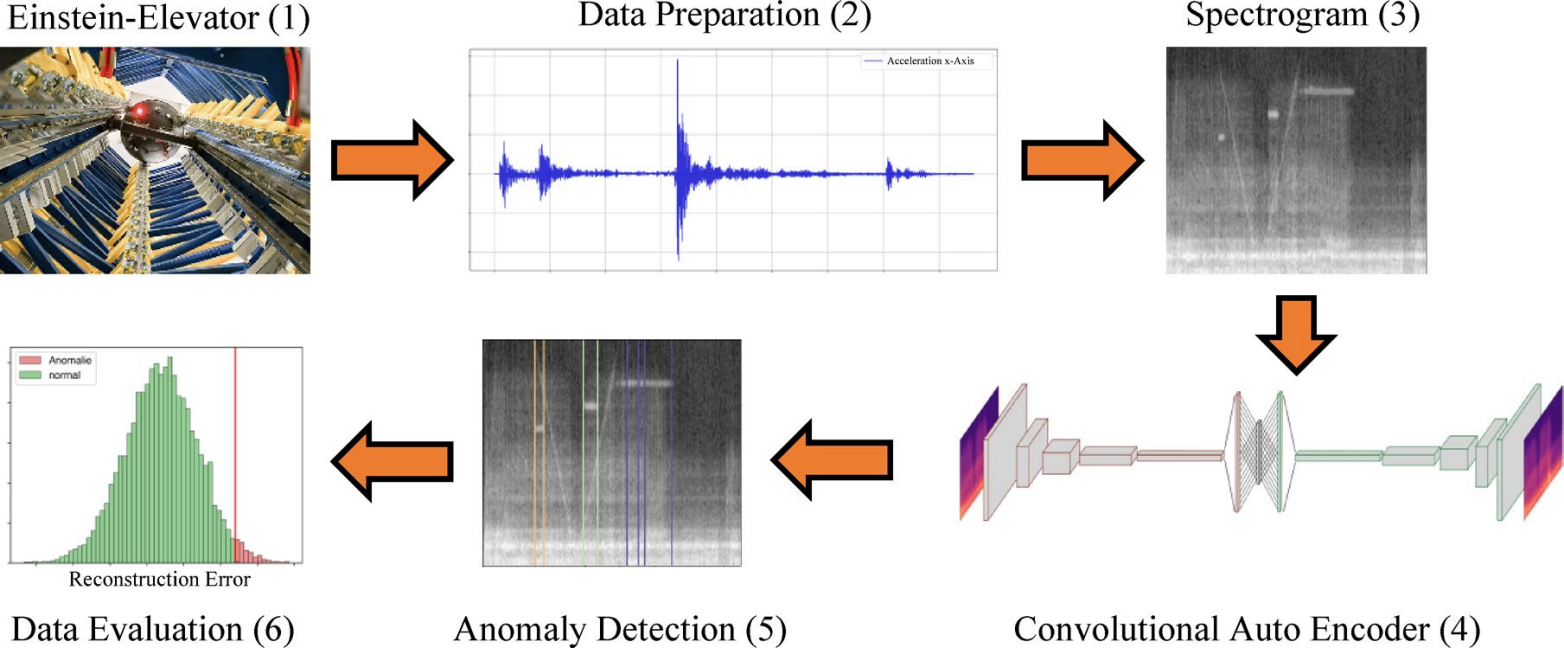
Steps for the condition monitoring framework.

The EE’s CMS framework involves six steps (Fig. 4):

1. Recording of acceleration data on the EE during the zero-gravity phase.
2. Filtering and preprocessing of the measurement data.
3. Conversion of the data from the time domain to the time–frequency domain using a Short-Time Fourier Transform (STFT).
4. Use of the resulting spectrograms to train the convolutional autoencoder (CAE).
5. Testing the trained CAE with artificially created anomaly samples.
6. Evaluation of the autoencoder with regard to its reconstruction error.

A total of 1800 flights have been carried out since the EE went into action in 2019 until this study started. These flights are divisible in operational test flights such as testing the emergency stop, simulations of Mars and Moon gravity, microgravity flights, as well as drop flights starting from the top of the EE. For this work, microgravity flights with a gravitational duration between 3.9 and 4.3 s were chosen as most of the experiments will be conducted under these conditions. Resulting, out of the 1800 flights a data set of 484 flights were taken for the condition monitoring framework. For the 484 flights, the selection of training data is limited to acceleration in the x-direction, which will be explained more later. The database allows flights to be filtered based on the duration of the gravitational phase. Furthermore, in the data preprocessing step, all spectrograms are standardized based on the shortest test duration, so that they have a uniform format for processing in the model. Duplicate timestamps in the measurement data are filtered out. The spectrograms are limited to frequencies between 0 and 2500 Hz, as this is where most frequencies occur.

10% of the flights will be used as validation data, while the remaining 90% will be divided into training and test data. The aim is to develop a model that correctly classifies both normal and faulty flights. The sensor data collected from the zero-gravity experiments with the EE must therefore first be pre-processed.

### Data preparation

**Fig. 5:**
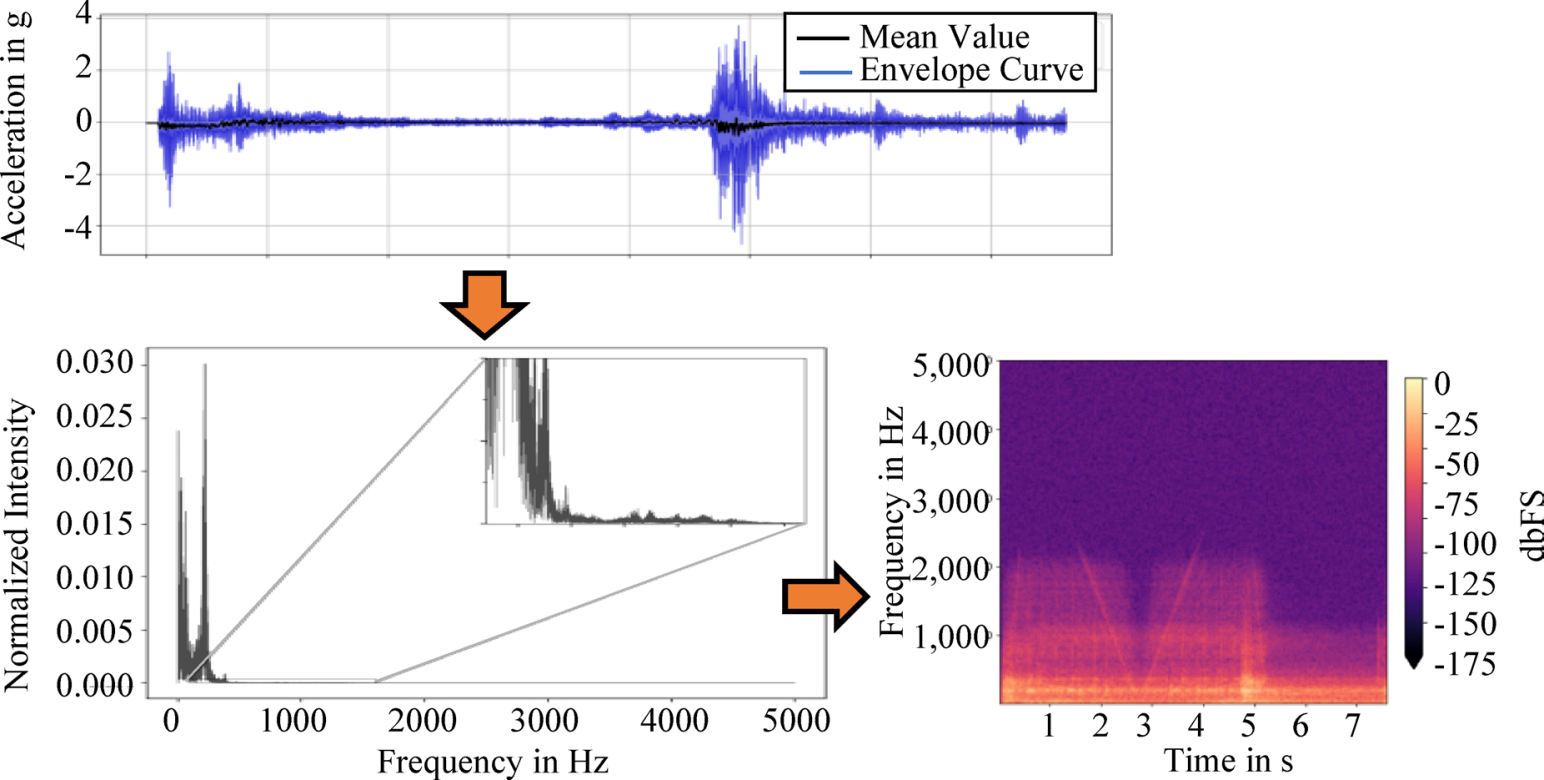
Fast Fourier Transformation of the acceleration data into frequency data with spectrogram visualization, dbFS stands for decibel relative to full scale.

The pre-processing of the flight data uses the Python libraries Numpy and Pandas to efficiently clean the data. First, irrelevant data points are removed in standby mode, resulting in cleansed data frames with relevant acceleration data. The data visualization helps to understand the vibration trajectories in x- and y-direction, which mark important transitions during the flight experiment, such as the initial acceleration and deceleration phase. To interpret the data correctly, a frequency analysis is performed using FFT showing that most of the oscillations occur in the 0–400 Hz range as shown in Fig. 5.

Errors such as erroneous measurement peaks and duplicated data points are corrected by filtering and interpolation so that the data sets are more reliable. Non-representative flight samples are excluded automatically to reduce the variance in the data set and refine the training data selection. The flight data is processed sequentially to avoid memory bottlenecks. The resulting cleaned data sets are stored in a linked database structure that allows easy access to measurement data and parameters and provides the basis for subsequent modeling approaches.

The main objective of the database system is the clear preparation of flight samples for systematic data processing. Three interconnected databases are created: two for the processed acceleration plus the velocity data and one central feature database, that contains the flight parameters and enables to filter the flight samples for an ideal training data selection. The flights are processed in a Python script and entered into the databases. The data preparation process validates the format of the data. The database entries include key phases and parameters of a flight, such as the start and end of the gravitational phase and its duration.

A sample object is created for each flight including raw and cleaned measurement data. The parameters are used to decide whether the flight is relevant. Relevant data is added to the feature database, taking into account characteristics in the time and frequency domain. This database-supported system enables the pre-classification and filtering of flight samples for further analysis within the framework. The successful integration of the linked database structure into an online database enables seamless access to the comprehensively pre-processed flight data. This infrastructural expansion allows the entire database to be systematically searched and relevant measurement data to be accessed in a targeted manner. After the sample object has been created, the parameters are used to check whether the flight phases represent the flight profile according to Fig. 2 or whether the flight data is irrelevant due to errors or unusable flight configuration, for example.

As mentioned before, the accelerations in the x and y directions are very similar. The vibrations in the z-direction, on the other hand, are superimposed by the imposed travel profile. The assumption here is that this makes it more difficult to recognize vibration patterns due to errors in z-axis data. Since occurring undesired vibration couple in all three axes, the selection of training data is therefore limited to the acceleration in the x-direction, neglecting the information from the y-direction, which is considered redundant. This was done for simplification purposes, although a decoupling of the x- and y-direction may provide further information and point to further anomalies.

The frequency distribution over time is analyzed using spectrograms based on a STFT, which mainly considers frequencies in the range of 0–2,500 Hz since most frequencies occur there. To ensure consistency in input size for the autoencoder, all selected spectrograms are resized to match the shortest flight duration observed within the filtered dataset. This standardization allows the spectrograms to be stored as uniform black and white 8-bit png-images. The database system filters unimportant or irrelevant data, particularly those not representative of microgravity conditions. It makes it possible to exclude flights with duplicated time stamps and to classify the flight data according to the duration of the microgravity phase. Anomaly detection is approached in the context of a semi-supervised learning method, as it is assumed that only normal operating conditions are present due to the novelty of the system. This approach is described in more detail in the following section.

### Model development for the CMS

**Fig. 6:**
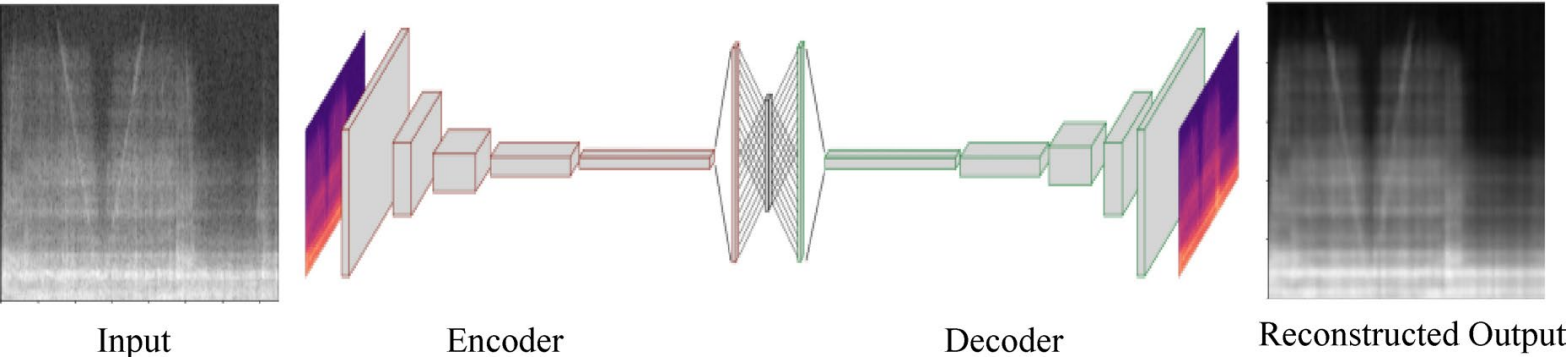
An abstract representation of a convolutional autoencoder.

After processing and selecting the data, a model is developed for evaluating the collected sensor data in order to analyze the machine status within the CMS. As the measurement data from the acceleration sensors was converted into frequency images using a STFT, a convolution neural network (CNN) is a suitable structure, as it is able to effectively recognize and process local patterns and spatial hierarchies in the frequency images, i.e., spectrograms. This property makes it possible to deal particularly well with the image-like structures of the frequency spectra and to efficiently extract relevant features for classification or analysis. For this purpose, an anomaly detection model is implemented in form of a convolutional autoencoder (CAE), as shown in Fig. 6.

A convolutional autoencoder is trained to compress a high-dimensional input into a low-dimensional vector representation comprising only the most inherent features of the input. Based on these derived features, the input is reconstructed into the output yielding a reconstruction error as a deviation measurement of the current sample from the imposed normal model behavior. Flight samples of the EE can be categorized by identifying faulty patterns via this reconstruction error. The anomaly threshold can be set based on the distribution of reconstructed pixel deviations inside the training data set. For the exact localization of anomalies, a difference matrix between input and output is analyzed with a sliding window. An anomaly is present if each pixel column in the window has at least one value above the threshold value. At the end of the analysis, contiguous anomaly areas are determined. In the original spectrogram, the time intervals classified as faulty by the model are automatically highlighted with vertical boundaries of random colors. The size of the window influences the detection accuracy, and the optimal choice of window size and threshold values is crucial for the performance of the algorithm.

Since the data must be normalized for training the deep learning model, the image pixels are transformed to the value range from zero to one. In addition, the total amount of data is divided into training and test data in order to first train and then test the model.

In model development for the CM, the main focus is on optimizing the architecture of a CAE for anomaly detection using the tuner module of the Keras library. This optimization is done by systematically adjusting various hyperparameters to find the best possible model structure.

The optimized parameters include the number of layers in the encoder and decoder part of the model. In the encoder, the size of the feature maps is reduced by adjusting the step length of the convolution. The convolution steps are optimized dynamically. Dropout layers are inserted to avoid overfitting, whereby both the frequency and the rate of the dropout are adjusted. In the decoder, the compression is reversed by transposed convolutional layers to ensure that the output has the same dimension as the input. To avoid odd dimensions of the feature maps, padding layers are used to expand the input data to the nearest power of two, while cropping layers restore the original dimensions in the output. Another important aspect of optimization is the number and size of the filters in the convolutional layers. The number of filters is increased in a specified range and determined by the tuning, as are the dimensions of the filter matrices. The bottleneck of the model, which represents the latent dimension, is realized via fully linked layers. Here too, the number of units in the bottleneck is adjusted to optimize model performance. In addition, the choice of activation functions within the layers is investigated in order to achieve the best results for anomaly detection. The CAE was tuned using the specified reference data set in a multi-stage process. In the first step, both the type of parameters and their rough ranges were determined for further tuning. In the second tuning step, 50 tuning operations were performed with random variation of the identified tuning parameters shown in Table 1. The chosen values for each tuning parameter are marked.

**Table 1:**
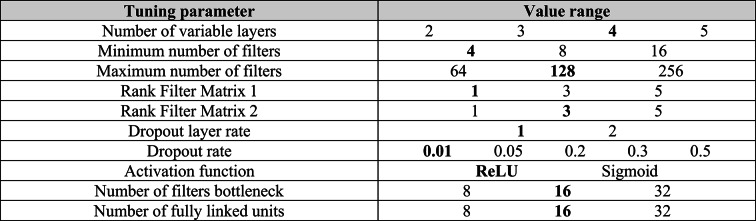
Tabular overview of the tuning parameters and the ranges in which they were varied. In addition, the optimal parameter values are marked in black.

**Fig. 7:**
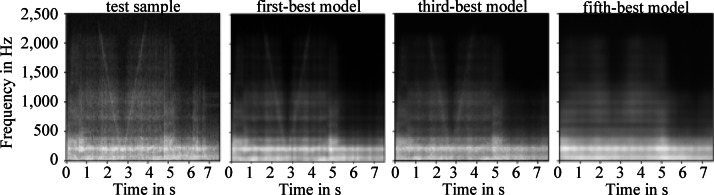
An exemplary test sample of the EE is shown on the left. Next to it, from left to right, the reconstructions by the first, third and fifth best model of the model tuning are shown.

To visualize the influence of model tuning, Fig. 7 shows first an exemplary test sample of the EE on the left site, and continues with the reconstructions of the best, third-best and fifth-best model of the model tunings for the test sample from left to right. The selection of these models from fifty in total was driven by their optimized parameter configurations, such as activation functions, number of layers, dropout rate, and filter matrix size, which aligned well with the data set’s characteristics, leading to improved reconstruction quality and robust model performance.

## Results

For the structured and comparable analysis of parameter variations of the condition monitoring framework, a reference data set is created based on existing data that includes 484 flights of the EE. These flights are limited to a gravitational duration of 3.9 to 4.3 s and represent microgravity samples. It is assumed that a model that can reconstruct this data is also able to reproduce spectrograms of deviating flight configurations. This assumption relies on the similarity of the characteristic structures in the spectrograms. As mentioned before, 10% of the data set is allocated for validation purposes, while the remaining 90% is divided in a 70:30 ratio for training and testing. This strategy is essential to assess model performance and mitigate the risk of overfitting.

For a quality- and quantity-based evaluation of anomaly detection, the model with the lowest reconstruction error has to be determined. A central evaluation criterion is the correct classification of the flight data. Since there are no labeled faulty flights, anomalies are generated and the classification is based on the reconstruction error using an anomaly threshold. The threshold value and the localization of anomalies are examined during the evaluation by varying relevant parameters. The accuracy of the model to detect anomalies is defined as:

3 $$acc = \frac{{n_{tp} + n_{tn} }}{{n_{dA} + n_{eA} }}$$

where n_tp_ and n_tn_ are the numbers of true positive and true negative classified anomalies and n_dA_ and n_eA_ are the numbers of the detected anomalies and existing anomalies. Moreover, the precision, recall, and F1-score have been calculated.

### Evaluation of anomaly detection

**Fig. 8:**
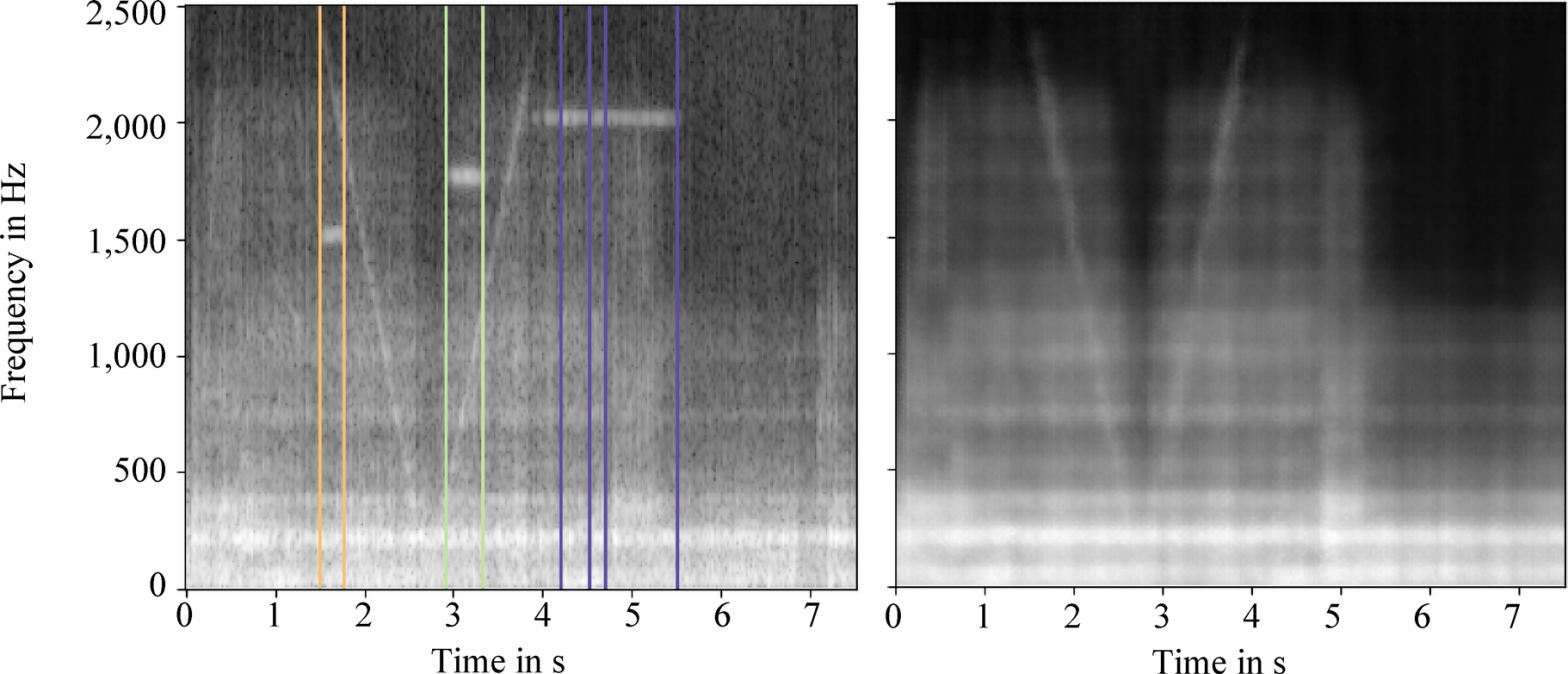
Comparison of the input spectrogram (left) and the reconstructed one (right). The four anomalies have not been reconstructed; however, the anomaly at 2000 Hz was falsely divided into two different anomalies decreasing the accuracy of the model (false-negative anomaly).

Out of the relevant 484 flight samples, 48 samples were modified with artificial created anomalies to evaluate the model. Moreover, those data should proof that the proposed model is able to recognize anomalous samples in general and the anomalous frequency patterns inside the spectrogram. In this connection, the anomalies were implemented randomly in the form of stochastic noise, as specific failure modes and their resulting anomalies are not yet known. In this context, different features of the anomalies were examined, such as their localization inside the spectrograms as well as their intensity, number and shape to verify the ability of the model. For this purpose, the model has learned to reconstruct the spectrograms of the ideal condition of the system, so that anomalies as deviations are not reconstructed. Those anomalies are highlighted by the system itself. Regarding the implementation of multiple anomalous patterns into a frequency range between 500 and 2350 Hz, the model was able to detect anomalies with a probability of 48.5%. The reason for this low accuracy is that in the most cases large anomalies are not classified as a single one but as multiple anomalies as shown in Fig. 8.

By limiting the presence of artificial anomaly patterns for analysis to frequencies ranging from 50 to 1,000 Hz, which also covers the range with a high frequency density, the probability of detection decreases to 23.8%. Increasing the intensity of synthesized anomalous patterns with a factor of 20 increases the accuracy to 50.2%. However, a further increase of the intensity has no influence on the detection rate of the model. The reason for this is that depending on their place of occurrence, anomalies stand out differently caused by the varying intensities of the spectrograms, especially in the area of low frequencies. As a result, an accurate and unified anomaly threshold for all anomalies is difficult to derive for the model.

Another subject of examination is the possibility of the model to detect single anomalies within a flight sample. Thereby, it was possible to increase the intensity of the anomalies based on their location. Using an intensity factor of 5000 for anomalies occurring at frequencies with f < 500 Hz (highest frequency density) as well as a factor of 20 for f > 500 Hz, the detection increased to a value of 81%. As a consequence, a threshold based on the average reconstruction error could be derived.

**Fig. 9:**
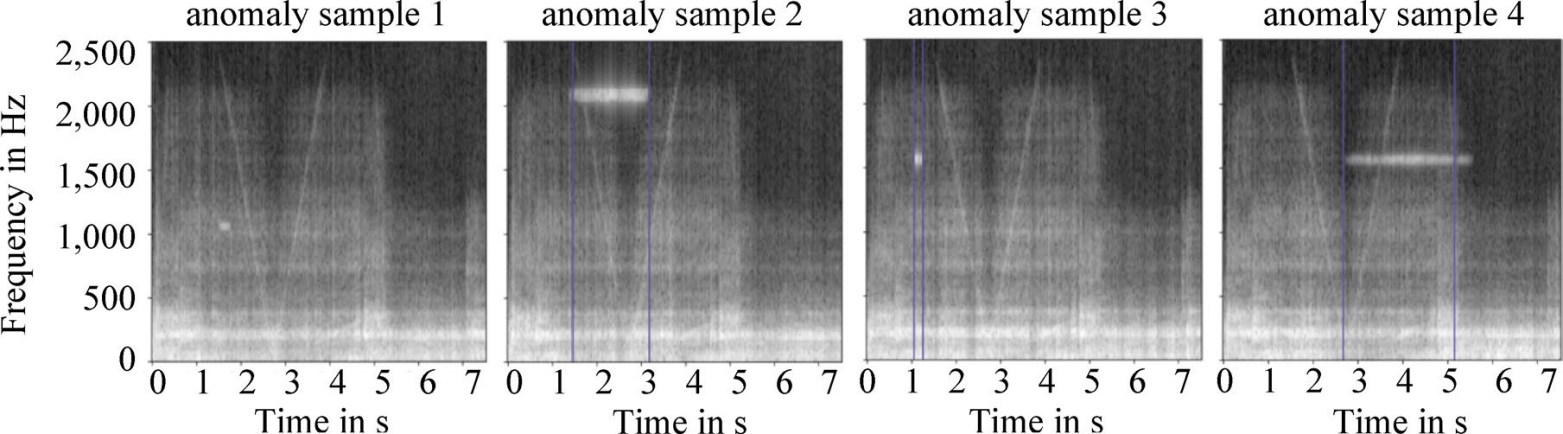
Various shapes of examined anomalies and detection behavior of the model.

Using the same conditions regarding single anomalies, also their shapes were varied (Fig. 9) by changing the bandwidth (increasing the height of the spots in the spectrograms) or their duration (increasing the width of the spots in the spectrograms). With respect to the primarily mentioned variation of the anomalies, smaller bandwidths influence the probability of detection negatively (58%), whereas anomalies with larger bandwidths don’t have a major influence concerning their detection by the model. The manipulation of the duration of an anomaly has also an effect on the detection. Based on the same circumstances, narrow anomalies (0.2–0.3 s) are better detectable (91%) than wider ones starting at 1.5 s (46%). A possible explanation for this is that, due to the statistical behavior of the anomaly patterns, the wider the anomalies the higher the possibility of magnitudes below the threshold. Consequently, the model splits up those anomalies into multiple ones.

**Fig. 10:**
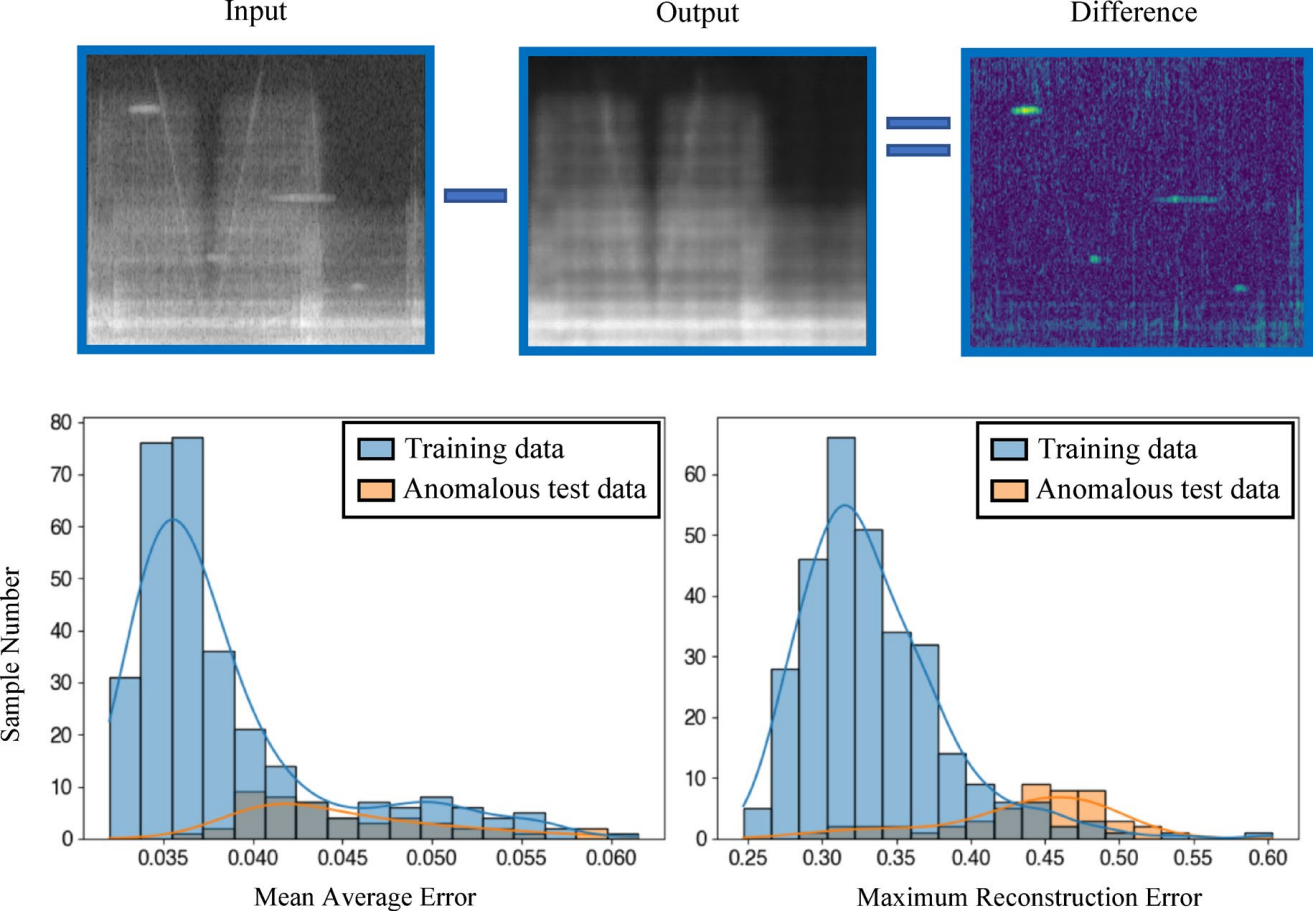
The input spectrogram (top left) and the reconstructed one (middle). The difference of both is seen in the top right of the picture. Using the MRE, anomalies are detected easier in comparison to the MAE.

The localization of anomalies is possible in a limited manner. Nevertheless, a further investigation was made to analyze if the model is able to classify anomalous and normal flight samples. For this, the maximal reconstruction error (MRE) was used for the evaluation in contrast to the former use of the mean average error (MAE). In Fig. 10, the error distribution of the anomalous samples shifted to the right using the MRE in comparison to the usage of the MAE. However, the differences are not high enough to achieve a total classification due to the assumption that the model is overfitted to the majority of training samples. Consequently, even some of the normal samples, which are slightly different, have high reconstruction errors.

All in all, the performance of the current CAE-model is highly depending on different features of the anomalies, especially on their intensity. The major reason for this is a high basic error resulting from the variable operating states of the facility and the resulting overfitting to a certain spectrogram type of the model.

### Use of data augmentation

Neural networks tend to under-/overfit in cases with small numbers of training data. In our case overfitting can be observed based on the former described results. This means that new unknown data can no longer be mapped within the model. The model is therefore no longer able to adapt to different circumstances and contexts. It has memorized the properties of the training data set and reacts poorly to new data. There are various strategies for avoiding this and adapting the generalization capability. In addition to the pure data expansion of images by scaling, mirroring or rotating, this includes, for example, the use of dropout zones in this paper. These offer the advantage of hardly any increased computational effort compared to L1 and L2 regularizations, which prevent overfitting and improving model generalization. Here, a section of the image is removed during training so that the dependency on hidden data is reduced (so-called cutout method). This results in an increased robustness of the network, as it is forced to learn the context of noisy data more accurately^34^. This prevents models from only learning the noise, for example.

**Fig. 11:**
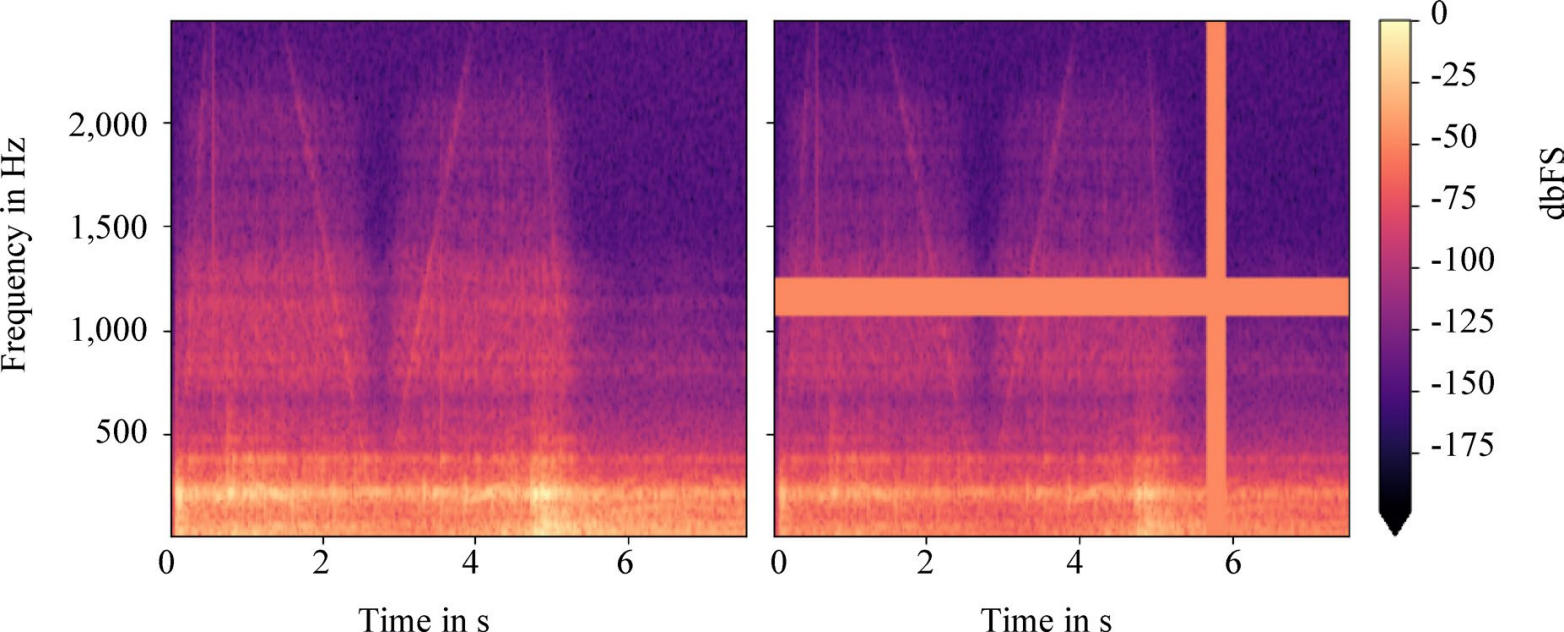
Time and frequency masking of an exemplary training sample (left) to create a new noisy sample (right).

Enrichment of the data set is achieved by masking the time and frequency axes. These approaches can be applied directly to the spectrogram images of the CAE. For this purpose, frequency masking (f_m_) consecutive rows or time masking (t_m_) consecutive columns of the image matrix of the spectrogram are replaced by the mean value of the intensity of the spectrogram. The values of f_m_ and t_m_ are generated from a continuous uniform distribution of values between zero and a maximum. In order to perform frequency or time masking within the interval [f_m0_, f_m0_ + f_m_] or [t_m0_, t_m0_ + t_m_], the starting point f_m0_ or t_m0_ must also be generated from a continuous uniform distribution in the range [0, t_m,max_—t_m_] or [0, f_m,max_—f_m_]. In the course of this work, a guideline according to Park et al. is followed, according to which a combined time and frequency masking is carried out for each flight sample of the training data^35^. A corresponding example is shown in Fig. 11.

**Fig. 12:**
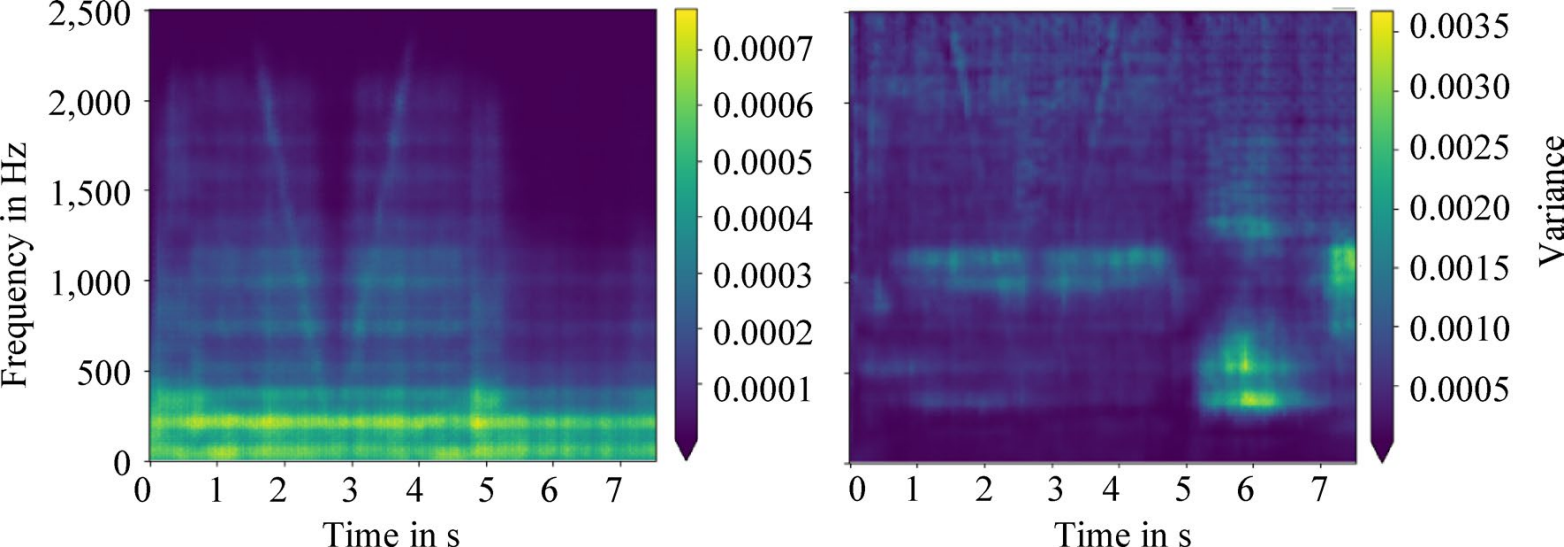
Averaged variance within the training reconstruction showing without (left) and with (right) extended training data set by time and frequency masking. The extended training data set increases the variance of the data.

The data generated in this way is used as additional training data and its influence on anomaly detection is investigated. The previous data set consisting of 484 flight samples was extended by 436 additional flight samples through data augmentation. As a result, 741 training samples in form of spectrograms are available for model training. The observed variances within the reconstruction of the training data are shown in Fig. 12. On the left side the variances are shown without and on the right side with the described data extension.

With an average variance of 1.5 × 10^–4^ to 5.85 × 10^–4^, the variance without data extension (left, Fig. 12) is approximately five times smaller than that with data extension (right, Fig. 12). In addition, the variance without data extension qualitatively follows the main characteristic intensity curves of the spectrograms. However, individual structures, occurring for instance with or after the deceleration phase (starting at 4 s), are not represented well. This suggests overfitting. After data enhancement, the existing deviations of the spectrograms within or after the deceleration phase are clearly better represented leading to improved generalization. Overall, the reproduction quality of the autoencoder is rated as satisfactory.

**Fig. 13:**
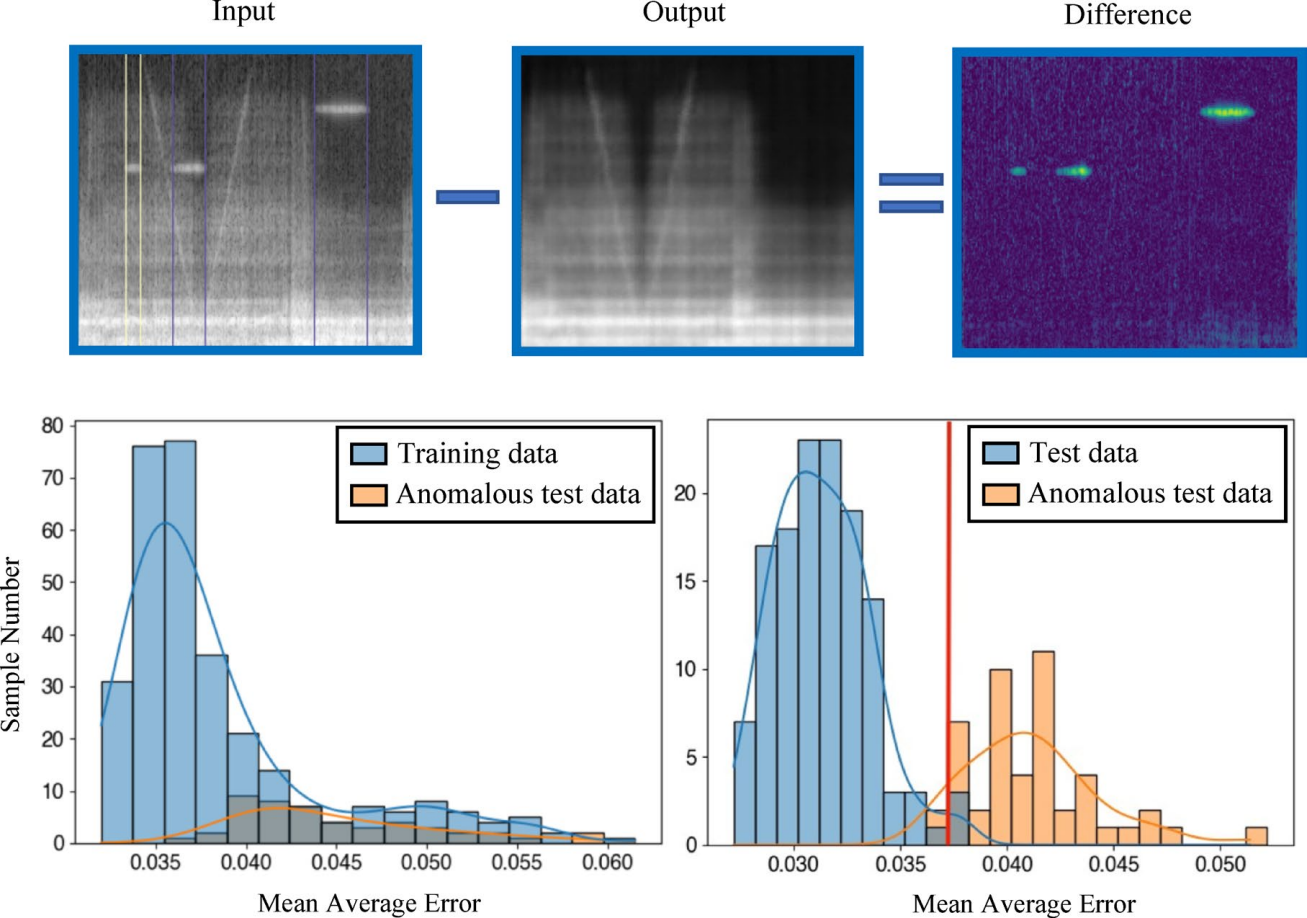
The input spectrogram (top left) and the reconstructed one (middle). The difference of both is seen in the top right of the picture. Using data augmentation and the test data as reference (down left), anomalous data improves the detection accuracy (down right).

When the distribution of the mean reconstruction error of the test data is plotted, Fig. 13 shows that the basic error of the model decreases due to the data expansion, which reduces the mean value of the error distribution. Anomalies are therefore much easier to detect and a threshold of 0.037 can be chosen. With this, the identification quality is 97.22%, whereas before the accuracy was strongly dependent on the shape of the anomalies. Based on this threshold, a comparison of the quality of the models presented here is possible by calculating the accuracy, precision, recall, and the F1-score (Table 2). In this context, it is seen that an overall improvement of the model is given with the use of data augmentation. Especially, the precision and the F1-score have improved. The better precision value indicates that the model has improved at identifying real anomalies and is less prone to false positive classifications. As a result, the F1-score, which is the harmonic mean of precision and recall, has also improved, as the recall remained high (changing only slightly). This suggests that a more balanced behavior of the model is given regarding identified anomalies and reducing false negatives.

**Table 2:** Metrics of the presented models.

Metrics	Model based on MAE in %	Model based on MRE in %	Model using DA in %
Accuracy	74.22	84.14	97.22
Precision	33.83	45.45	93.88
Recall	93.75	83.33	95.83
F1-Score	49.72	58.82	94.84

## Discussion

Based on the results, the proposed model is able to detect anomalies in general and highlight these. However, it is difficult to define a generally valid threshold for all anomalies. This is a consequence of the varying intensities and the resulting gradient inside a spectrogram. Here, dividing the spectrograms into suitable areas and examining them with different threshold values can be an approach for better detection of anomalies. Furthermore, the detection of anomalies increases significantly by using data augmentation. Still, both with data augmentation and without, this depends mainly on their shape and their area of occurrence and therefore their frequency. Also implementing a single anomaly in contrast to multiple anomalies, improves the ability of the model to classify the data correctly.

Nevertheless, one important point is the number of considered samples, which is in total 484 flights. Neural network models with a small size of data tend to over- or underfit, which was therefore also a challenge in our research case. Due to the fact that the MRE of the training data is higher than the one of the test data, the probability of overfitting based on some significant characteristics of the data is higher. Therefore, the model outputs higher MAEs and MREs regarding training samples which deviate slightly from the majority of these. As a consequence, the model is underfitted for theses variances. Furthermore, the influence of the small data basis is also shown by the positive effect caused by the use of data augmentation. Generally, data augmentation increases the number and variety of training data. As a result, the model becomes more robust and stable against those variations and the risk of overfitting is decreased. However, an analysis of the latent space of the model, which is the lower-dimensional space, can also be promising. For this, the CAE projects the essential features of the original in the latent space. Based on this projection, significant deviations can be identified from the distribution of normal data. Typical used methods to highlight those deviation in the latent space is the computing of the Mahalanobis distance or performing a cluster analysis.

It should be noted that synthetic anomalies are not the same as real anomalies. Real anomalies can change in their manifestation, which can only be recognised or displayed via the continuous course of the system monitoring. Despite that, the CAE model is able to recognize anomalies which is fundamental for further studies with the aim to improve it considering real anomalies. However, no definitive statement can be made about its capabilities regarding real anomalous data, as this kind of data is missing. In this context, it is crucial to validate the model with those, so that more accurate statements can be made. Although the transformation of the time series data into spectrograms and thus images is helpful and a standard investigation tool in microgravity research, the process can lead to information loss. Consequently, very slight changes of the system would then not be represented and the detection of these changes would occur later. For this, time-series based neural networks such as LSTMs must be considered so that a transformation of the data is not needed. Especially, as LSTMs are able to remember long-range dependencies. However, due to this ability, they are also computationally intensive and require large data sets which can be a limitation in our current, small data set. Besides LSTM, Generative Adversarial Networks (GAN) have shown significant promise in generating high-quality synthetic data. This can be beneficial in augmenting small data sets. The adversarial nature of GANs allows for the generation of highly realistic anomalies, providing a robust means to test anomaly detection systems. Nevertheless, GANs also have their downsides, such as the potential instability during training. In addition, they require careful tuning of hyperparameters. In comparison, CAEs offer simplicity and are less computationally demanding. These aspects make them suitable for scenarios where computational resources are limited. Evaluating these models in the context of the EE’s unique conditions offers opportunities for further enhancing our CMS framework.

Independent of these, a combined analysis with other sensors, such as the acceleration sensors of the CCU, can give even more information about the state of the system. With this, a dependency between the microgravity quality and disturbances variables due to vibrations recorded by the PMU can be developed.

In summary, a successful application of a condition monitoring framework (shown in Fig. 4) was achieved for a complex system like the EE. For the iterative optimization loop between the time–frequency domain converted data, the training of the convolutional autoencoder, its testing with artificially created anomaly samples and the evaluation of the reconstruction error (steps 3 and 6 of the described framework), results showed that a 7^th^ step of data augmentation has to be added to create a sufficient amount of data and a sufficient quality of the model.

## Conclusion & outlook

Neural networks and thus machine learning is increasingly finding their way into technical systems. As the field of machine learning techniques is rapidly changing, also new approaches, techniques and models are developed. However, those are seldomly investigated with respect to unique, complex and new systems with a small amount of training data. This paper covers these two subject areas with regard to the Einstein-Elevator. Therefore, based on the state of the art, a framework for a CMS was developed and applied on the EE. In this context, we identified that the accelerometer of the PMU is the most promising sensor for the detection of anomalies at the EE by developing a CMS. The framework developed includes both the pre-processing of sensor data and the creation and optimization of a data-driven model for diagnosing faulty operations. Based on the fact that spectrograms are a widely used tool for frequency analysis, a CAE was developed to detect anomalies out of these. Therefore, the implemented autoencoder was trained to reconstruct spectrograms of normal flight samples. Subsequently, anomalies should not be reconstructed. The model was evaluated based on the size of the reconstruction error depending on the used sample. For this, faulty flight data was artificially generated. The reconstructions of the anomalous spectrograms have shown that the autoencoder does not reconstruct injected frequency patterns. Thus, it only models the normal operating behavior. Nevertheless, the noise inside of the reconstructed samples was still high, so that anomalous samples could not be detected satisfiable. An overfitting was observed due to the small dataset. To counteract overfitting, an approach for expanding the training data using time- and frequency-dependent masking of the spectrograms was implemented. With this, anomalies were detectable with an accuracy of approximately 97%. Moreover, additional metrics such as the recall or the F1-score have demonstrated a positive performance of the model. Finally, the results of this study were used to improve the procedure of the development for a general framework.

In the future, although the results seem promising, the model behavior still has to be examined considering real anomalies. Especially, the detection of minor changes within the spectrograms which result from the initial onset of wear can be challenging. For this, test beds of relevant components of the EE such as the roller bearings can be developed. Identified frequencies which are generated during fatigue tests can be inserted into the spectrograms and used as anomalous data for the CAE. As a result, the model can be examined with more realistic anomalies based on the recorded data of the test beds. Moreover, further options should be considered in order to increase the amount of training data and thus the mappable variance of normal operating conditions. In this context, this study predominantly employed cutout and time/frequency masking techniques for data augmentation. To further enhance the generalizability of our condition monitoring system, future research should include a comparative analysis of various data augmentation methods. One option would be a synthetic oversampling, where data is generated to increase the representation of rare classes. Another option is the use of noise injection, which involves adding random noise to training samples to increase model robustness. These methods can offer additional benefits, as they have shown promise in other domains by diversifying training datasets. Besides, they improved the adaptability of models to new and unseen data. Consequently, in combination with our approach, this can lead to an overall improved and a more robust system capable of handling a wider variety of anomalous patterns. By systematically comparing these different augmentation strategies, we can determine the most effective methods for applying to complex, small data environments like the EE. This exploration would provide valuable insights into the strengths and limitations of different augmentation strategies. It also significantly contributes to the development of a more universally applicable framework for condition monitoring across varied technical systems. Future work will also involve a comparative analysis of LSTM and GAN models to determine the optimal approach for anomaly detection in small data environments. With this, the significance and the margins of error of the models can then be calculated for a comparison.

With respect to the EE, future topics are the usage of further measurands, such as the x- and y-direction accelerations, and the combination of further data generated by the already existing sensors. Moreover, the usage of acoustic recordings should be considered, as an additional monitoring method. In this context, it is conceivable that the increase in training data and the consideration of additional data will also facilitate the localization of anomalies. The use of so-called evolutionary algorithms can help to optimize the design of an artificial neural network. Moreover, the framework should be extended with other analyzing methods, especially regarding the latent space. Also, an extension considering the implementation of alternative machine learning techniques enables the possibility of a multi-perspective framework that can be used for different kinds of data and therefore machines. In this context, the developed model should also be tested with similar complex systems.

In conclusion, the presented research shows, that the application of a general framework for complex systems which includes the implementation of a CMS was successful. The CMS is used to detect slight changes and to avoid unintentional shut downs of the system. This is particularly necessary for systems with high requirements, as is the case with drop towers and their microgravity quality due to vibrations induced during the acceleration phase of the experiment. In addition, the framework with the databases generally enables a detailed overview of the system’s condition, which also increases the understanding of its general behavior. In order to increase the information gain from the available data even more, the framework should be extended to other sensors of the system and machine learning approaches, so that later on, the framework can also be used universally for a variety of areas where a CMS is required. Besides, the identification and quantification of wear parameters and their influence on the condition parameters such as the microgravity quality is a long-term aim of the presented work.

## Data Availability

The data sets used and/or analyzed during the current study are available from the corresponding author or the co-authors on reasonable request.

## Declarations

### Author’s Statement

**Special Unit**

*g* = Earth’s gravity (1 *g* = 9.81 m/s^2^). It is written in italics to avoid confusion with the unit gram (“g”, not written in italics).
